# Sjögren's Syndrome and Devic's Disease: A Synchronised Saga

**DOI:** 10.7759/cureus.51763

**Published:** 2024-01-06

**Authors:** Saket Toshniwal, Jiwan Kinkar, Yatika Chadha, Sourya Acharya, Sunil Kumar

**Affiliations:** 1 Medicine, Jawaharlal Nehru Medical College, Datta Meghe Institute of Higher Education and Research, Wardha, IND; 2 Neurology, Jawaharlal Nehru Medical College, Datta Meghe Institute of Higher Education and Research, Wardha, IND; 3 Psychiatry, Jawaharlal Nehru Medical College, Datta Meghe Institute of Higher Education and Research, Wardha, IND

**Keywords:** neurological manifestation, autoimmune overlap, sjogrens syndrome, neuromyelitis optica, devics disease

## Abstract

Devic's disease, commonly known as neuromyelitis optica (NMO), is a rare relapsing autoimmune illness of the central nervous system. Occasionally, it is associated with other autoimmune diseases such as Sjögren's syndrome (SS). Dry mouth and dry eyes are two symptoms of SS, a chronic autoimmune condition marked by inflammation and dysfunction of the exocrine gland. While SS primarily affects the exocrine glands, it can also manifest with a range of extraglandular features, including neurological manifestations, as in our case where the patient initially presented with neurological symptoms and was diagnosed with NMO. Owing to the persistent relapses along with sicca symptoms that occurred late, SS was diagnosed on further evaluation. Although the association of SS with NMO is not very common, the initial presentation of SS with neurological symptoms, as in our case, is what makes it more unique.

## Introduction

Sjögren's syndrome (SS) is a chronic autoimmune disorder characterised by dysfunction of the exocrine gland and can be associated with extraglandular manifestations [1]. Neurological manifestations such as loss of sensation, motor weakness, movement disorders, and aphasia, including neuromyelitis optica (NMO) and neuromyelitis optica spectrum disorders (NMOSD), are seen in approximately 20-25% of diagnosed SS cases. Although it is uncommon for neurological manifestations to be the initial presenting feature of primary SS, as in our case [2], not many cases of this autoimmune overlap have been reported, and very few of them began with neurological symptoms in primary SS, which makes it the rarest and most complex saga [3]. The mechanism of central nervous system (CNS) involvement in SS is rather unclear; therefore, it is important to report such rare case scenarios to help consider the possibility of an autoimmune overlap and administer prompt treatment with early diagnosis. Autoimmune disorders and their clustering have been documented previously, and it is hence important to shed light on such scenarios for early and prompt evaluation [4].

## Case presentation

**Figure 1:**
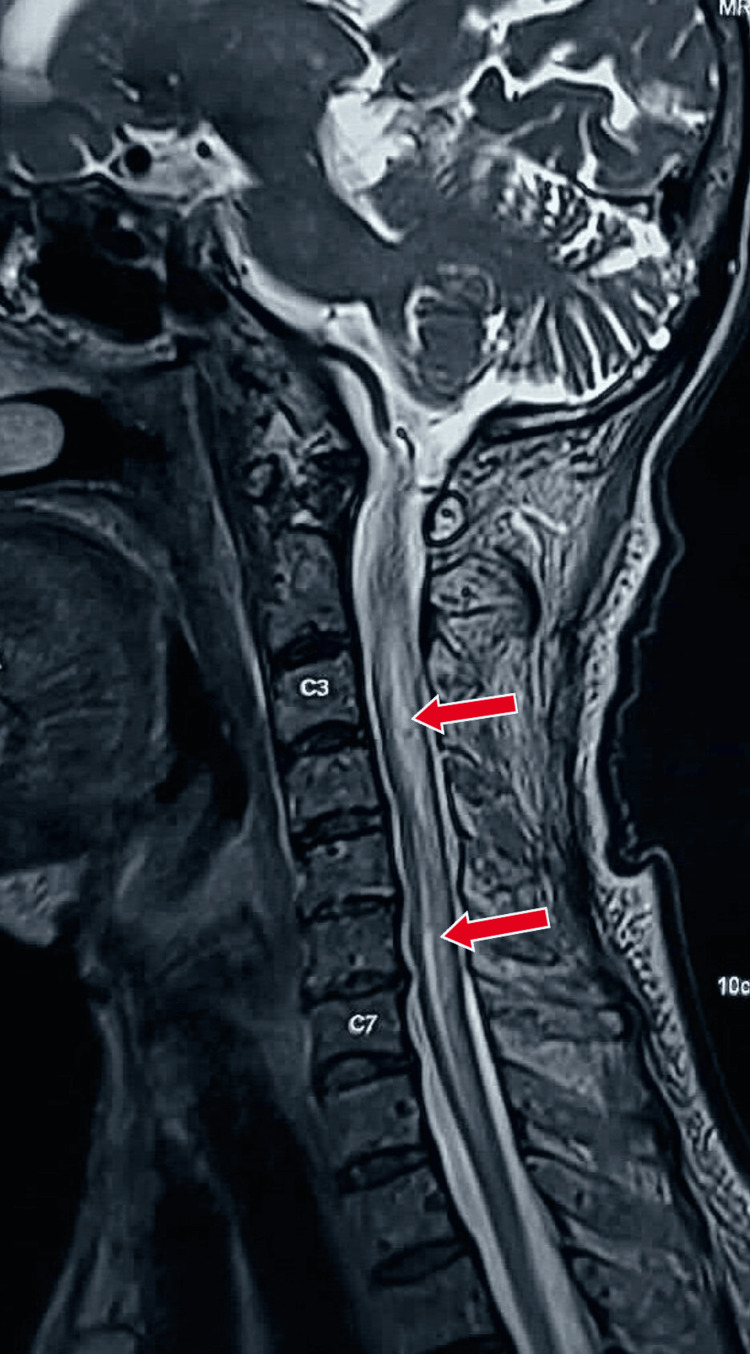
MRI cervical spine (T2 sagittal section) showing longitudinal extensive lesion of spinal cord at C2-D1 level with central cord prominence marked with red arrows. MRI: magnetic resonance imaging.

**Figure 2:**
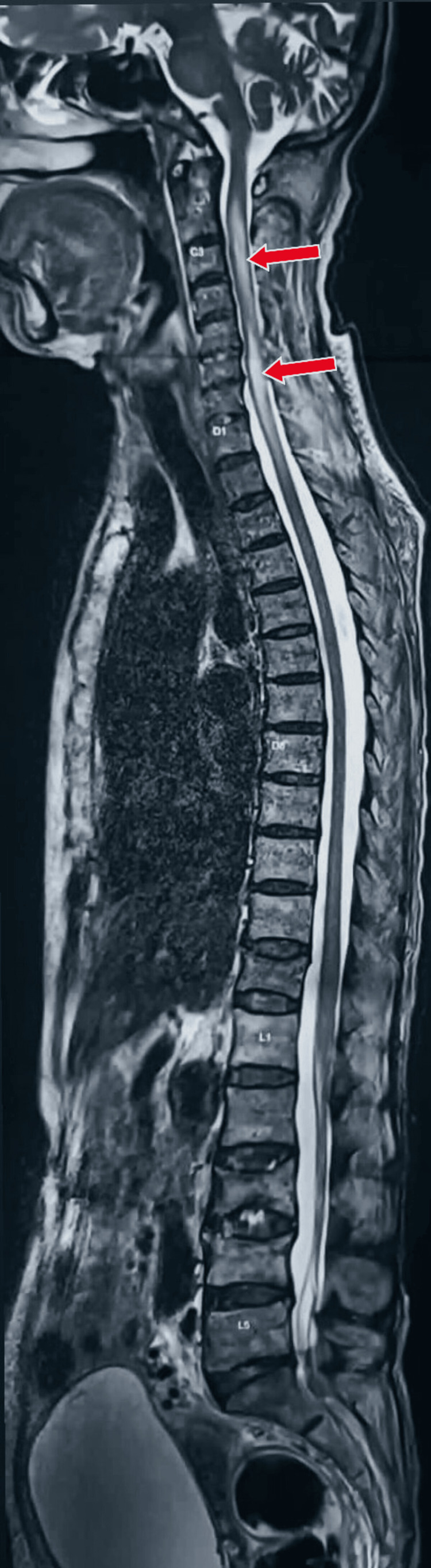
MRI whole spine screening (T2 sagittal section) showing longitudinal extensive lesion of spinal cord at C2-D1 level with central cord prominence marked with red arrows. MRI: magnetic resonance imaging.

A female patient in her late 30s, with no significant medical and family history, presented to the outpatient department with weakness in all four limbs, beginning with the upper limbs, for three months, which was progressive in nature. On examination, deep tendon reflexes in both upper extremities were found to be absent with diminished deep tendon reflexes in the lower extremities. Plantars were bilateral extensors. On sensory examination, there were diminished touch and vibration senses in both lower limbs up to the joints. Position sense was impaired in both great toes. Possibilities of subacute combined degeneration of spinal cord (SACD), transverse myelopathy, NMO, and copper myelopathy were kept as differential diagnoses, and the patient was investigated. On evaluation, magnetic resonance imaging (MRI) brain and cervical spine with whole spine screening with contrast was done, which was suggestive of a normal study on MRI brain screening and showed a longitudinal extensive lesion of the spinal cord with central cord prominence at over three contiguous vertebral segments on MRI cervical spine with whole spine screening, as shown in Figures 1, 2.

Cerebrospinal fluid (CSF) examination revealed extensive pleocytosis (>50 leukocytes/microlitre) with the absence of oligoclonal bands, as shown in Table 1.

**Table 1:** CSF analysis. CSF: cerebrospinal fluid.

CSF laboratory parameters	Results	Reference range
Protein	70 mg/dL	15-60 mg/dL
Sugar	76 mg/100 mL	50-80 mg/dL
Cells	65 cells per cu. mm	0-8 cells per cu. mm
Lymphocytes	85%	70%
Neutrophils	15%	0-2%
Oligoclonal bands	0 (negative)	>3 (positive)

A seropositive status for the anti-aquaporin 4 immunoglobulin G antibody (AQP4-IgG) was established, which led to the diagnosis of NMO. The patient was treated with intravenous methylprednisolone (1 g/day) for three days followed by oral corticosteroids (tablet prednisolone 40 mg once a day to be continued) and immunosuppressants (tablet azathioprine 25 mg twice a day to be continued). The patient had a relapse of the same symptoms within a span of a month in spite of ongoing treatment. On further detailed history-taking and on leading questions, she complained of dry mouth and persistent burning eyes suggestive of dry eyes for more than a year which she was not well aware of. An ophthalmological examination revealed a positive Schirmer’s test bilaterally. Further immunological evaluation owing to the positive Schirmer's test revealed seropositive antinuclear antibody (ANA) with speckled pattern on immunofluorescence and a strong positive anti-SS-related antigen A (SS-A) and recombinant-52 (Ro-52) antibody which lead to the diagnosis of SS. The patient was treated with intravenous methylprednisolone (1 g/day) and immunosuppressants (tablet azathioprine 25 mg twice a day) which was continued with no further relapsing symptoms. After a six-month follow-up of our patient, she is in good health and is able to carry on her daily chores with no relapsing neurological manifestation except the sicca symptoms persist.

## Discussion

The association between SS and NMO highlights the complex interplay between autoimmune diseases and their potential impact on the central nervous system. Recent research has shown that SS and NMO share common immunopathological mechanisms, suggesting a potential link between these two conditions. Studies have found that patients with SS have a higher prevalence of 10-20% in NMO-IgG AQP-4 antibody-positive patients compared to the general population [5]. Hence, SS patients are more prone to acquiring NMO and vice versa. This association of SS and NMO in synchrony also increases the episodes of relapses of either of the conditions, owing to poor response to treatment [6].

Additionally, patients with SS and NMO have been found to have similar clinical features and disease courses, making it harder to diagnose. A detailed history by asking leading questions to the patient with regard to symptoms becomes an important aspect to help diagnose the primary condition, as in our case, where the patient was unaware of her sicca symptoms.

The diagnosis of SS is based on a combination of clinical, serological, and histological criteria. Clinical features include dry eyes, dry mouth, and evidence of exocrine gland dysfunction. Serological tests, such as the presence of anti-SSA and anti-SSB antibodies, can support the diagnosis. Histological examination of salivary gland biopsies may also show lymphocytic infiltration. In the case of NMO, the diagnosis is based on clinical criteria, including the presence of optic neuritis and myelitis, as well as supportive criteria, such as MRI evidence of spinal cord lesions and serological evidence of NMO-IgG, as in our case, which fulfilled the criteria by Wingerchuk et al. for NMO. The diagnosis of SS was made by fulfilling the American College of Rheumatology (ACR) and European League Against Rheumatism (EULAR) criteria [6,7].

In order to reduce symptoms and stop the disease from progressing, SS and NMO are managed using a multidisciplinary approach. Artificial tears and saliva replacements are used in the treatment of SS with the goal of easing symptoms like dry mouth and eyes. Immunosuppressive treatment might be required in more serious situations. In order to lower inflammation and stop relapses, immunosuppressive medications, including corticosteroids and immunomodulatory drugs such as azathioprine and mycophenolate mofetil, are commonly used in the treatment of NMO. In refractory situations where frequent relapses occur despite receiving standardised immunotherapies and a poor response to therapy, plasma exchange may also be taken into consideration [8]. The prognosis of SS and NMO varies depending on the severity of the disease and the response to treatment. With appropriate management, many patients with SS can achieve symptomatic relief and maintain a good quality of life. However, SS is a chronic condition that requires long-term monitoring and follow-up. Similarly, NMO is a relapsing disease, and regular monitoring is essential to detect relapses and adjust treatment accordingly.

To sum up, the correlation between SS and NMO is an intriguing field of study that emphasises the intricacy of autoimmune disorders and their possible influence on the central nervous system. It is critical for the proper diagnosis, course of treatment, and long-term care of NMO patients to acknowledge this relationship and take other autoimmune comorbidities into consideration. In order to create better treatment that can enhance patient outcomes, more research is required to comprehend the fundamental mechanisms underlying these illnesses with such rare overlap syndromes [9].

## Conclusions

SS in association with NMO and its spectrum disorder suggests shared immunopathological mechanisms between these conditions. Close collaboration between rheumatologists and neurologists is crucial for the diagnosis of such rare overlaps for better management and follow-up of patients with SS and NMO. The identification of other autoimmune disorders associated with NMO emphasises the need for a comprehensive evaluation of patients with NMO to optimise treatment strategies and improve patient outcomes. Detailed history-taking and leading questions regarding symptoms to help diagnose the primary pathology have to be given equal importance and should not be ignored.
